# Successful Outcome of Acute Promyelocytic Leukemia Complicated by Bone Marrow Necrosis and Posterior Reversible Encephalopathy Syndrome, During Treatment With an All-Trans Retinoic Acid Plus Arsenic Trioxide-Based Regimen

**DOI:** 10.7759/cureus.104972

**Published:** 2026-03-10

**Authors:** Theodora M Venou, Maria Mainou, Konstantina Voura, Ester S Mbonde Mouangue, Nicolaos Flaris, Efthymia Vlachaki

**Affiliations:** 1 Second Department of Internal Medicine, Hippokration General Hospital of Thessaloniki, Thessaloniki, GRC; 2 Laboratory of Anatomic Pathology, Hippokration General Hospital of Thessaloniki, Thessaloniki, GRC; 3 Second Department Of Internal Medicine, HIppokration General Hospital of Thessaloniki, Thessaloniki, GRC

**Keywords:** acute promyelocytic leukemia, all-trans retinoic acid, arsenic trioxide, blood marrow necrosis, posterior reversible encephalopathy syndrome (pres)

## Abstract

Acute promyelocytic leukemia (APL) is a distinct subtype of acute myeloid leukemia characterized by the *PML-RARA* fusion gene and frequent coagulopathy. All-trans retinoic acid (ATRA) plus arsenic trioxide (ATO)-based regimens achieve excellent complete remission rates exceeding 95% with durable molecular responses. However, treatment-related and disease-related complications may occur during therapy. We report a case of a 57-year-old man with APL complicated by bone marrow necrosis, pulmonary complications, and posterior reversible encephalopathy syndrome. Multidisciplinary supportive care with continuation of ATRA-ATO therapy resulted in complete hematologic and molecular remission. This case highlights the importance of recognizing and managing complex complications in APL while maintaining curative therapy.

## Introduction

Acute promyelocytic leukemia (APL) is a distinct and highly treatable subtype of acute myeloid leukemia (AML), characterized by the reciprocal translocation t(15;17)(q22;q21), resulting in the formation of the promyelocytic leukemia-retinoic acid receptor alpha (PML-RARA) fusion gene. This genetic alteration disrupts normal myeloid differentiation and confers a unique biology and clinical profile [1]. The introduction of all-trans retinoic acid (ATRA) and arsenic trioxide (ATO) has revolutionized treatment outcomes, with current chemotherapy-free ATRA/ATO regimens achieving complete remission rates approaching 100% in non-high-risk patients [2]. Despite these advances, APL remains a hematologic emergency due to complex coagulopathy involving both hemorrhagic and thrombotic complications, with thrombosis occurring in 4.5-8.8% of patients [3].

Bone marrow necrosis (BMN) is a rare but severe complication in hematologic malignancies, histologically defined as necrosis of hematopoietic tissue and medullary stroma with preservation of cortical bone. It typically presents with systemic symptoms such as bone pain, fever, pancytopenia, and elevated markers of cell turnover [4,5]. Although more commonly reported in AML and acute lymphoblastic leukemia (ALL), BMN has rarely been described in APL and may be underdiagnosed due to overlapping clinical features and diagnostic challenges [3,6,7]. BMN can occur both at initial diagnosis (reported in 2.4% of AML and 3.2% of ALL patients) and as a consequence of intensive leukemia therapy [3]. Mechanisms contributing to BMN are multifactorial and may involve leukemic infiltration, cytokine-induced vascular injury, treatment-related toxicity, and microvascular occlusion, especially in the context of disseminated intravascular coagulation (DIC) or endothelial dysfunction. Notably, DIC is a common complication in acute promyelocytic leukemia, occurring in approximately 60-90% of patients at diagnosis [4,8].

In addition to BMN, patients with APL may develop a range of serious treatment-related complications. These include differentiation syndrome, a potentially life-threatening inflammatory reaction associated with ATRA and ATO therapy [9], and pulmonary complications, including infection and infarction, which may mimic invasive fungal disease but are often thrombotic in nature, especially in the context of DIC [10]. More specifically, differentiation syndrome results from rapid differentiation and activation of leukemic cells, leading to a systemic inflammatory response and increased capillary permeability. Clinically, it may present with fever, dyspnea, pulmonary infiltrates, weight gain, pleural or pericardial effusions, hypotension, and renal dysfunction. Management requires prompt initiation of systemic corticosteroids (e.g. dexamethasone) and, in cases of marked leukocytosis or severe clinical manifestations, cytoreduction to decrease the leukemic cell burden, along with appropriate supportive care [11].

Posterior reversible encephalopathy syndrome (PRES) is a clinicoradiological syndrome characterized by acute neurological symptoms-most commonly headache, seizures, altered mental status, and visual disturbances-associated with vasogenic edema predominantly affecting the posterior cerebral white matter on neuroimaging, and typically reversible with prompt recognition and management of the underlying cause [12].

Herein, we describe a rare and diagnostically complex case of APL complicated by DIC, extensive BMN, differentiation syndrome, PRES, and pulmonary infarction. This case highlights the intricate interplay between disease biology, treatment toxicity, and systemic complications and underscores the need for high clinical vigilance and adaptive management strategies throughout the therapeutic course.

## Case presentation

A 57-year-old male presented to the Emergency Department with dyspnea and a recent diagnosis of deep vein thrombosis (DVT) involving bilateral iliac veins. The patient had a three-month history of progressive bilateral lower limb edema leading to the detection of extensive DVT and subsequent pulmonary embolism (PE). He had already been on fondaparinux at a therapeutic dose for nine days. His past medical history was significant for bipolar disorder. The family history was negative for any hematologic disorders or malignancies.

Upon admission, the patient was hemodynamically stable. Physical examination of the heart, lungs and abdomen was unremarkable. Initial laboratory findings revealed pancytopenia, characterized by anemia (Hb 8.4 g/dL), neutropenia (500/μL), and thrombocytopenia (115,000/μL). Coagulation studies showed hypofibrinogenemia (123 mg/dL), prolonged clotting times, and markedly elevated D-dimer levels (23,200 ng/mL), indicative of disseminated intravascular coagulation (DIC). Biochemical analysis demonstrated mild hyperuricemia and increased lactate dehydrogenase (LDH). A detailed summary of all laboratory findings at admission is provided in Table 1.

**Table 1:** A detailed summary of all laboratory findings. The first column presents the laboratory values at the time of admission (day 1), while the second column shows the corresponding values on the day the patient developed bone marrow necrosis (day 5). APTT: activated partial thromboplastin time, INR: international normalized ratio.

Parameters	Admission	At Bone Marrow Necrosis	Reference Range	
White Blood Cell Count (WBC)	3,900/μL	18,100/μL	4,000-11,000/μL	
Neutrophils	500/μL	16,000/μL	2,000-7,500/μL	
Lymphocytes	800/μL	1,300/μL	1,000-4,800/μL	
Monocytes	2,700/μL	0	200-800/μL	
Hemoglobin (Hb)	8.4 g/dL	6.5 g/dL	12-16 g/dL	
Hematocrit (Hct)	23%	18.50%	36-46%	
Platelets (PLT)	115,000/μL	11,000/μL	150,000-450,000/μL	
Glucose	161 mg/dL	275 mg/dL	70-110 mg/dL	
Urea	50 mg/dL	104 mg/dL	15-40 mg/dL	
Creatinine	1.08 mg/dL	1.23 mg/dL	0.6-1.2 mg/dL	
Uric Acid	7.9 mg/dL	4.1 mg/dL	3.5-7.2 mg/dL	
Calcium	9.5 mg/dL	9.2 mg/dL	8.5-10.5 mg/dL	
Phosphorus	3.8 mg/dL	3.1 mg/dL	2.5-4.5 mg/dL	
Potassium	4.7 mmol/L	3.7 mmol/L	3.5-5.0 mmol/L	
Sodium	138 mmol/L	136 mmol/L	135-145 mmol/L	
Total Proteins	6.9 g/dL	7.2 g/dL	6.0-8.3 g/dL	
Albumin	4.5 g/dL	4.6 g/dL	3.5-5.0 g/dL	
Alkaline Phosphatase (ALP)	73 U/L	73 U/L	40-150 U/L	
Total Bilirubin	0.7 mg/dL	0.7 mg/dL	0.1-1.2 mg/dL	
Direct Bilirubin	0.9 mg/dL	0.9 mg/dL	0.0-0.3 mg/dL	
Magnesium	2.63 mg/dL	2.6 mg/dL	1.7-2.5 mg/dL	
Amylase	26 U/L	30 U/L	30-110 U/L	
Troponin	13 pg/mL	191 pg/mL	<14 pg/mL	
Aspartate Aminotransferase (AST)	18 U/L	467 U/L	10-40 U/L	
Alanine Aminotransferase (ALT)	15 U/L	154 U/L	7-56 U/L	
Lactate Dehydrogenase (LDH)	347 U/L	1388 U/L	140-280 U/L	
Creatine Phosphokinase (CPK)	59 U/L	458 U/L	20-200 U/L	
Gamma-Glutamyl Transferase (GGT)	59 U/L	390 U/L	8-61 U/L	
C-reactive protein (CRP)	30.8 mg/dL	325 mg/dL	<0.5 mg/dL	
Procalcitonin	0.21 ng/mL	0.38 ng/mL	<0.05 ng/mL	
INR	1.21	1.96	0.8-1.2	
APTT	45.7 sec	34.1 sec	25-35 sec	
Fibrinogen	123 mg/dL	402 mg/dL	200-400 mg/dL	
D-Dimers	23,200 ng/mL	33,741 ng/mL	<500 ng/mL	

**Figure 1:**
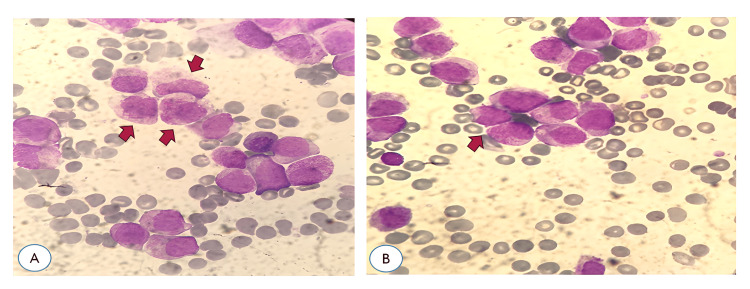
Bone marrow smear stained with May–Grünwald–Giemsa, ×100 magnification (oil immersion). Increased number of atypical promyelocytes are observed, showing abundant granular cytoplasm and the presence of Auer rods. The morphologic features are consistent with acute promyelocytic leukemia (APL). Red arrows indicate representative cells displaying these characteristic morphologic features.

A peripheral blood smear identified blasts with Auer rods, raising suspicion for APL. On the first day of hospitalization, an urgent bone marrow aspirate and trephine biopsy were performed, which demonstrated blasts of similar morphology in the bone marrow. Figure 1 shows a representative bone marrow smear at ×100 magnification stained with May-Grünwald-Giemsa.

**Figure 2:**
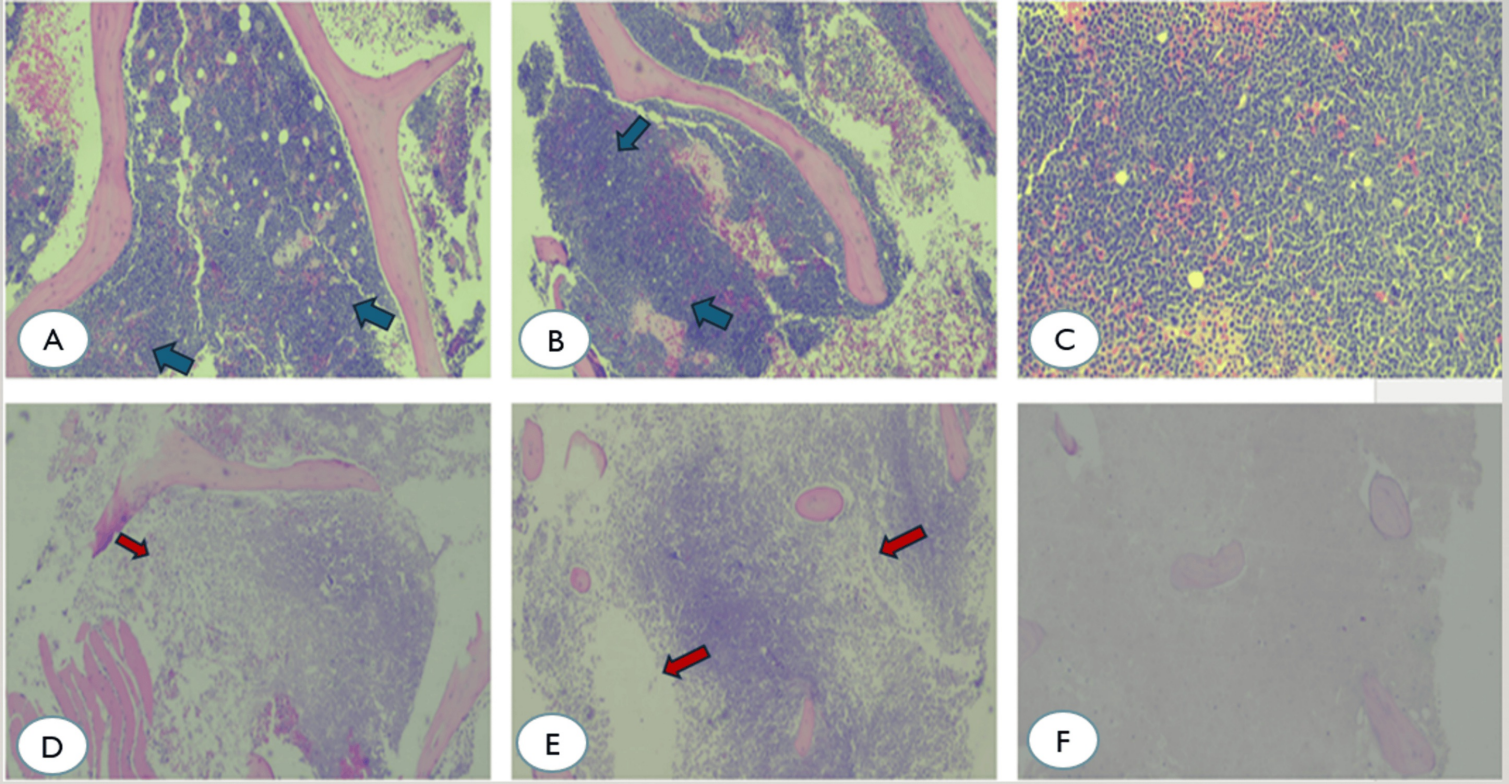
Dense infiltration of the bone marrow biopsy specimen by abnormal promyelocytes. (A-C) Dense infiltration of promyelocytes characterized by their large size, high nuclear-to-cytoplasmic ratio, and finely granular cytoplasm (1st row). (D-F) Representative histopathological images of bone marrow necrosis (BMN) showing extensive necrosis affecting 70% of the biopsy, with residual marrow demonstrating 50% cellularity, marked hemosiderin deposition, and trilineage hematopoietic maturation (2nd row) (hematoxylin–eosin staining, x10). Blue arrow points out sites of dense infiltration of the bone marrow by abnormal promyelocytes, while red arrows indicate sites of hypocellular bone marrow.

These findings are typical of APL and reflect extensive marrow involvement at diagnosis (Figure 2).

Given the hematological abnormalities and coagulopathy, treatment with an ATRA was initiated on day two of admission at a dose of 45 mg/m²/day. Supportive care included transfusions of blood products, including cryoprecipitate, platelets, and packed red blood cells, to correct coagulopathy, anemia, and thrombocytopenia. Therapeutic-dose fondaparinux for DVT management was continued. Three days after admission, the presence of the *PML-RARA* fusion gene was confirmed on molecular testing. Patient was classified as low risk APL based on white blood cell count on admission (<10,000/μl), and ATO (0.15 mg/kg IV) was added to the treatment regimen according to current protocol.

By day five of hospitalization, the patient developed weight gain, fever and leukocytosis (WBC 13,000/μl), raising concern for differentiation syndrome, a known complication of ATRA therapy. Hydroxyurea was initiated to control leukocytosis, and corticosteroids (dexamethasone 10mg iv, every 12 hours) to mitigate this risk. On day nine, during the fourth dose of ATO administration, he developed severe systemic pain, refractory to opioid analgesia. Repeat laboratory analysis revealed worsening anemia (hemoglobin: 6.5 g/dL), severe thrombocytopenia (platelets: 11,000/μL), and a sharp increase in white blood cell count (18,100/μL), with significant neutrophilia (neutrophils: 16,000/μL). The dose of fondaparinux was adjusted based on the platelet count. Specifically, therapeutic anticoagulation was typically continued when platelet counts were above ~50×10⁹/L, modified or supported with platelet transfusions when counts were 30-50×10⁹/L, and temporarily held only when counts fell below ~30×10⁹/L. 

Additionally, a marked elevation in LDH (1388 U/L) was noted, suggesting extensive cell turnover or tissue damage. Marrow aspiration revealed a gel-like, brown material, and microscopic examination showed amorphous necrotic tissue with scattered remnants of destroyed marrow cells. Bone marrow biopsy confirmed extensive ischemic necrosis affecting 70% of the marrow, consistent with BMN.

The full spectrum of laboratory abnormalities at this stage is detailed in Table 1. Moreover, representative images of the BMN are presented below, illustrating the characteristic histopathological changes (Figure 2-2nd row). Given the severity of BMN, ATRA-ATO therapy was discontinued, along with anticoagulation therapy due to low platelets. The patient received intensive supportive care, including transfusions and broad-spectrum antimicrobial therapy. Once clinically stabilized, one cycle of chemotherapy was resumed with ATRA and idarubicin (day 15), substituting ATO due to its suspected role in BMN.

**Figure 3:**
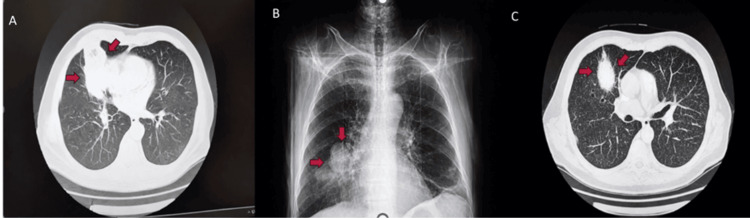
Multiplanar thoracic imaging demonstrating a persistent right paracardiac pulmonary infiltrate. Imaging findings raised a differential diagnosis of fungal versus bacterial infection; due to concern for systemic dissemination from necrotic tissue, a right middle lobectomy was performed (day 56). (A and C) Axial view and (B) Coronal view. Red arrows highlight the areas of infiltration in each image.

During induction therapy with idarubicin (12 mg/m² IV) and ATRA (day 26), the patient developed febrile episodes, and imaging revealed a persistent right paracardiac pulmonary infiltrate, raising a differential diagnosis of fungal versus bacterial infection (Figure 3).

Despite prolonged administration of broad-spectrum antibiotic and antifungal therapy for one month, the pulmonary lesion showed no sign of resolution. Given the absence of an identified pathogen and the persistence of elevated inflammatory markers, bronchoscopy was performed, which was inconclusive, and a thoracic surgery consult suggested pulmonary infarction. Bronchoalveolar lavage (BAL) was performed to investigate the pulmonary lesion and the suspicion of fungal infection. Cytological examination of the BAL fluid showed 0-1 leukocytes and 1-2 erythrocytes per high-power field. Microbiological culture of the BAL specimen was positive for fungi and specifically yielded *Candida glabrata.* Molecular testing for *Mycobacterium tuberculosis *complex by PCR was negative. In addition, Ziehl-Neelsen staining did not reveal acid-fast bacilli, and mycobacterial cultures using both Löwenstein-Jensen medium and the MGIT (mycobacteria growth indicator tube) system were negative.

Due to the increased risk of systemic infection from necrotic tissue, a right middle lobectomy was performed after the completion of induction therapy and the restoration of hematologic parameters (day 56). Postoperatively, the patient showed gradual improvement, with stabilization of respiratory function and resolution of systemic inflammatory markers. Moreover, following induction therapy, the patient received intrathecal injection of methotrexate, cytarabine and corticosteroids. He was discharged from the hospital while quantitative real-time PCR confirmed a significant reduction in *PML-RARA* transcripts. At diagnosis, the *PML-RARA* transcript level was initially measured at 5.81 × 10⁻¹. Following induction therapy, a significant reduction was observed, with the level dropping to 3.45 × 10⁻⁵.

**Figure 4:**
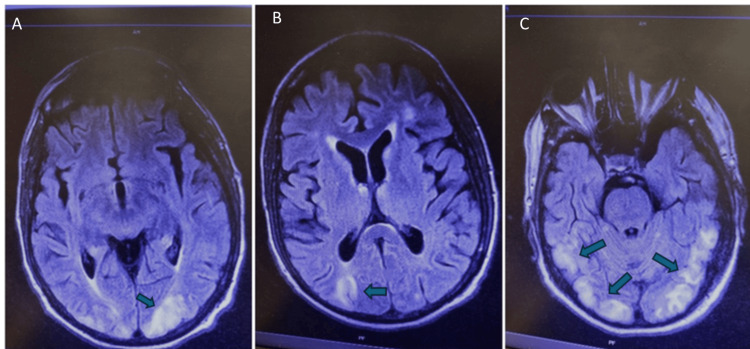
Axial brain MRI (T2/FLAIR). (A-C) Images are showing bilateral parieto-occipital hyperintense areas in the white matter, consistent with posterior reversible encephalopathy syndrome (PRES) during consolidation therapy with ATRA and ATO (day 100). Images are displayed without a scale bar. Blue arrows in the figure indicate the hyperintense areas in the white matter.

However, during the first consolidation with ATRA plus ΑΤO (day 100), the patient developed hypertension and diplopia, followed by a generalized tonic-clonic seizure. Brain MRI revealed areas of increased MR signal on T2/FLAIR sequences within the brain's white matter (Figure 4).

Clinical and imaging findings were consistent with PRES. Antihypertensive (amlodipine, olmesartan) and antiepileptic therapy (valproic acid, levetiracetam) were initiated, leading to stabilization and symptom resolution.

ATRA-ATO was resumed (day 107) with a brief pause, and the patient achieved remission. Quantitative detection of *PML-RARA* transcripts became undetectable following the first consolidation cycle (day 153), confirming molecular remission. The patient is currently seven months after completion of the third consolidation cycle and is undergoing scheduled three-monthly surveillance with bone marrow evaluation and *PML-RARA* molecular monitoring, with findings consistent with ongoing complete remission.

## Discussion

This case underscores the multifactorial complexity of acute promyelocytic leukemia (APL) and highlights BMN as a rare but serious complication arising during treatment. While APL is highly curable in the modern era with ATRA and ATO, its management remains nuanced due to potential life-threatening coagulopathy and treatment-induced complications. Our patient, who developed extensive BMN within the first 10 days of induction with ATRA-ATO, exemplifies the diagnostic and therapeutic challenges posed by this entity.

BMN is defined as the necrosis of hematopoietic tissue and medullary stroma with preservation of the cortical bone. It is histologically characterized by an amorphous eosinophilic background, ghost cells, and loss of marrow architecture. The reported incidence of BMN in acute leukemias ranges from 2% to 3%, with higher frequencies in AML of monocytic subtypes and ALL [3,5]. While more commonly reported at diagnosis, BMN can also emerge during treatment or relapse, as seen in our case and others [8,10].

The pathogenesis of BMN remains poorly understood, but proposed mechanisms include leukemic infiltration, cytokine-mediated apoptosis, immune complex deposition, and, particularly, microvascular failure. ATRA and ATO have been implicated in endothelial injury and microthrombosis, contributing to marrow ischemia. Cull et al. reported a case where early exacerbation of coagulopathy during ATRA therapy led to BMN, hepatic dysfunction, and acute kidney injury [9]. Similarly, arsenic-induced apoptosis of endothelial cells and inhibition of angiogenesis have been suggested as contributing factors [8].

Our case supports the hypothesis of treatment-related microvascular damage, as BMN developed in the context of elevated LDH, worsening cytopenias, and severe pain without overt DIC. In contrast to previous fatal cases, our patient’s BMN was reversible following withdrawal of ATO and treatment adjustment. This mirrors findings from Ianotto et al., where a case of focal, non-lethal BMN resolved despite continuation of therapy, emphasizing that not all BMN indicates treatment failure [13].

BMN also presents diagnostic challenges. Cytopenias, bone pain, and elevated LDH, it's classic triad-are non-specific and may mimic disease progression, infection, or differentiation syndrome. As emphasized by Harada et al. and Ranathunga et al., bone marrow aspiration often yields a dry tap or jelly-like material, necessitating repeated aspirations or biopsy [6,7]. MRI may be supportive, showing diffuse geographic signal changes with peripheral hypointense bands, but biopsy remains the gold standard [7]. Immunohistochemistry in necrotic samples can be misleading due to antigen degradation or non-specific antibody binding [6]. In some cases, as shown by Saito et al., Charcot-Leyden crystals may be present even without eosinophilia, potentially linked to local cytokine release such as Interleukin-1 beta (IL-1β), which further contributes to marrow injury [14].

Prognostically, BMN has traditionally been associated with poor outcomes, including lower complete remission (CR) rates and shorter overall survival. According to Badar et al., patients with acute myeloid leukemia (AML) who developed bone marrow necrosis (BMN) had a median overall survival of 3.7 months compared with 14 months in those without BMN [3]. However, more recent reports-including the present case and those described by Ianotto and Saito suggest that outcomes may be favorable when BMN is promptly recognized, appropriately managed, and the underlying disease responds well to therapy [13,14].

Finally, our case adds to the limited but growing evidence of BMN in APL patients receiving first-line ATRA-ATO, a setting where such necrosis is still underrecognized. It emphasizes the importance of vigilance for early symptoms of BMN-pain, cytopenia, refractory anaemia, and the need for multidisciplinary management, including hematopathology, radiology, infectious disease, and supportive care. A summary of previously reported BMN cases in acute promyelocytic leukemia, including those associated with APL, is provided in Table 2.

**Table 2:** Summary of Bone Marrow Necrosis (BMN) Cases in Acute Promyelocytic Leukemia (APL). This table summarizes key case reports of BMN in APL, detailing the type of leukemia, clinical findings, and patient outcomes [1,2,8-10,13,15-17].

Case Report	Type of Leukemia	Main Findings	Outcome
Limentani et al. (1994) [15]	APL	BMN after ATRA + hydroxyurea, with massive cell lysis	Fatal outcome due to marrow necrosis
Cull et al. (1997) [9]	APL	BMN associated with coagulopathy and ischemia	Fatal outcome despite supportive care
Zhang et al. (2000) [1]	APL	BMN after high-dose ATO; dose-dependent effect suggested	Relapse and death after partial remission
Ishitsuka et al. (2004) [8]	APL	BMN during reinduction with As2O3 + HU + 6-MP, extensive necrosis	Fatal outcome, refractory disease
Ianotto et al. (2010) [13]	APL	BMN during first-line As2O3; focal necrosis, non-lethal	Long-term remission achieved
Lakhwani et al. (2011) [2]	APL	BMN during ATRA induction, spontaneous resolution	Continued therapy, remission achieved
Hazenberg et al. (2021) [16]	APL	BMN after ATRA + ATO; severe pain, high LDH, confirmed by MRI and biopsy	Symptoms resolved after treatment interruption; therapy resumed and long-term remission achieved
Wang et al. (2024) [10]	APL	BMN in APL with novel TTMV::RARA fusion, CNS involvement	Achieved remission post-therapy but relapsed rapidly
Goodall et al. (2024) [17]	APL	ATRA-ATO-induced BMN with pain and pancytopenia	Patient survived with therapy modification

BMN is rarely diagnosed before death and is generally associated with poor outcomes [13]. However, in the setting of acute promyelocytic leukemia (APL) treated with ATO, nine pre-mortem cases have been reported. Among them, five patients died-three due to complications directly related to extensive BMN, such as sepsis, DIC, or multiorgan failure [8,9,15], while the remaining two died following disease relapse, with BMN not considered the primary cause of death [1,10]. The other four patients survived despite presenting with focal or partial necrosis [2,13,16,17]. Common presenting symptoms included bone pain and fever. These cases support the hypothesis that BMN may result from cumulative ATO exposure [13].

Another critical complication was posterior reversible encephalopathy syndrome (PRES), which presented during consolidation therapy with hypertension, diplopia, and seizure. PRES is an uncommon but recognized complication of ATRA-ATO therapy, thought to arise from endothelial dysfunction, cytokine activation, and loss of cerebral autoregulation [12]. To our knowledge, this is one of the few reported cases of PRES in a patient with acute promyelocytic leukemia. While PRES has been observed in various oncologic settings, including ALL and post-transplantation, as well as in AML, its incidence is very low. In our case, PRES developed during consolidation therapy with ATRA-ATO and was accompanied by hypertension and neurologic symptoms. Although causality cannot be firmly established, this observation highlights the need for clinical awareness and blood pressure monitoring in this patient population. This finding is consistent with the case reported by Creutzig et al., in which a five-year-old pediatric patient with standard-risk APL developed PRES, generalized seizures, and aseptic osteonecrosis during induction with ATRA-ATO therapy. Such cases underscore the importance of early recognition of neurologic signs and aggressive management during ATRA-ATO treatment in APL [18].

The patient’s persistent pulmonary infiltrate raised concern for invasive fungal infection, but was ultimately attributed to pulmonary infarction, confirmed after surgical resection. Pulmonary necrosis in APL is a rare but serious complication, often linked to thrombotic events associated with DIC [10]. Misdiagnosis as a fungal infection may lead to unnecessary antifungal exposure or delayed surgical management.

## Conclusions

This case illustrates the multifactorial complexity of APL treatment, particularly in patients presenting with severe thromboembolic disease, BMN, pulmonary necrosis, and PRES syndrome. The clinical course highlights the interplay between coagulopathy, leukemic burden, endothelial injury, and treatment-related effects, which together contributed to both systemic and organ-specific complications and necessitated an individualized, multidisciplinary approach. While BMN remains a poor prognostic factor in AML, its prognostic significance in APL appears less definitive, as remission may still be achieved with careful management despite severe complications. Further research is necessary to establish standardized therapeutic strategies for BMN in leukemia and optimize patient outcomes, recognizing that conclusions drawn from individual cases should be interpreted cautiously and viewed as hypothesis-generating rather than definitive.
